# Characterization of Mammalian ADAM2 and Its Absence from Human Sperm

**DOI:** 10.1371/journal.pone.0158321

**Published:** 2016-06-24

**Authors:** Heejin Choi, Sora Jin, Jun Tae Kwon, Jihye Kim, Juri Jeong, Jaehwan Kim, Suyeon Jeon, Zee Yong Park, Kang-Jin Jung, Kwangsung Park, Chunghee Cho

**Affiliations:** 1 School of Life Sciences, Gwangju Institute of Science and Technology, Gwangju, Korea; 2 The National Primate Research Center, Korea Research Institute of Bioscience and Biotechnology, Cheongju, Korea; 3 Department of Urology, Chonnam National University Medical School, Gwangju, Korea; University of Edinburgh, UNITED KINGDOM

## Abstract

The members of the ADAM (a disintegrin and metalloprotease) family are membrane-anchored multi-domain proteins that play prominent roles in male reproduction. ADAM2, which was one of the first identified ADAMs, is the best studied ADAM in reproduction. In the male germ cells of mice, ADAM2 and other ADAMs form complexes that contribute to sperm-sperm adhesion, sperm-egg interactions, and the migration of sperm in the female reproductive tract. Here, we generated specific antibodies against mouse and human ADAM2, and investigated various features of ADAM2 in mice, monkeys and humans. We found that the cytoplasmic domain of ADAM2 might enable the differential association of this protein with other ADAMs in mice. Western blot analysis with the anti-human ADAM2 antibodies showed that ADAM2 is present in the testis and sperm of monkeys. Monkey ADAM2 was found to associate with chaperone proteins in testis. In humans, we identified ADAM2 as a 100-kDa protein in the testis, but failed to detect it in sperm. This is surprising given the results in mice and monkeys, but it is consistent with the failure of ADAM2 identification in the previous proteomic analyses of human sperm. These findings suggest that the reproductive functions of ADAM2 differ between humans and mice. Our protein analysis showed the presence of potential ADAM2 complexes involving yet-unknown proteins in human testis. Taken together, our results provide new information regarding the characteristics of ADAM2 in mammalian species, including humans.

## Introduction

The a disintegrin and metalloprotease domain-containing protein (ADAM) family includes membrane-anchored proteins that share a conserved multidomain structure comprising an N-terminal signal sequence, a pro-domain, and metalloprotease, disintegrin, cysteine-rich, epidermal growth factor (EGF)-like, transmembrane, and cytoplasmic tail domains. The ADAM family members are widely distributed in different species and are present in a variety of tissues. At least 34 and 26 *ADAM* genes have been identified in mice and humans, respectively. More than half of the *ADAM* genes are known to be expressed exclusively or predominantly in mammalian male reproductive tissues, such as the testis or epididymis [1].

*ADAM2* was one of the first identified reproductive *ADAM* genes. Also known as PH-30 β or fertilin β, ADAM2 was originally identified as an integral membrane glycoprotein in guinea pig sperm. Analysis of guinea pig ADAM2 revealed that the protein is synthesized in testis and processed during sperm maturation. The proteolytic processing of ADAM2 during epididymal maturation of the sperm removes the pro- and metalloprotease domains, leaving the processed form with an N-terminal disintegrin domain [2, 3]. Transcripts for ADAM2 have been identified in the testes of numerous mammalian species, including mice, rats, rabbits, pigs, bulls, monkeys and humans [4–12].

Previous mouse knockout studies showed that male mice with deletions of *Adam2* or the closely related *Adam2* and *Adam3* are infertile, with their sperm showing defects during the fertilization process [13–16]. These knockout mice have provided insights into the remarkably complicated relationships between ADAM2 and the other ADAMs. For example, ADAM2 has been found to form diverse ADAM complexes in spermatogenic cells, including the ADAM1A-ADAM2, ADAM1B-ADAM2, ADAM2-ADAM3 and ADAM2-ADAM3-ADAM6 complexes [15, 17–19]. In addition, other ADAMs, such as ADAM4 and ADAM5, have been suggested to associate with ADAM2. Although ADAM7 is not believed to associate with ADAM2, these two ADAMs have been found to reciprocally regulate one another’s integrity [17, 20, 21]. The previous findings suggest that ADAM2 plays a central role in maintaining the stability of the proteins involved in the above-listed complexes. Moreover, complexes containing ADAM2 and ADAM3 have been shown to be important for various sperm functions in mice, including sperm-sperm aggregation, sperm-egg interactions and the movement of sperm from the uterus into the oviduct [13, 22–24].

In the present study, we investigated the mouse, monkey and human ADAM2 proteins. We found a relationship between the differential types of ADAM2 complex formation and a change in the cytoplasmic domain in mice. We generated specific antibodies against human ADAM2, and used them to analyze ADAM2 expression in monkeys and humans. The generated antibodies identified ADAM2 in monkey testis and sperm. We also identified ADAM2 (100 kDa) in human testis but not sperm. This suggests that ADAM2 has a different reproductive function in humans compared to mice and monkeys. This is the first characterization of human ADAM2 at the protein level.

## Materials and Methods

### Ethics statement

The biospecimens used in this study were provided by the Pusan National University Hospital; this hospital is a member of the National Biobank of Korea, which is supported by the Ministry of Health, Welfare and Family Affairs. All samples from the National Biobank of Korea were obtained with informed consent under institutional review board-approved protocols. The study of human sperm was also ratified through the Ethics Committee of Gwangju Institute of Science and Technology (GIST) and Chonnam National University (permit number: 20140818-BR-14-01-02). All participants signed an informed consent form permitting use of their semen remnants in this study.

### Animals

All procedures involving macaques (*Macaca fascicularis*) were approved by the Institutional Animal Care and Use Committee of Korea Research Institute of Bioscience & Biotechnology (KRIBB). The *Macaca fascicularis* testes and sperm were provided by the Korea National Primate Research Center, KRIBB. Each monkey was housed in an indoor individual cage [60 (W) × 80 (L) × 80 (H) cm^3^], fed commercial monkey chow (Harlan Laboratories, Indianapolis, IN, USA) supplemented daily with various fruits and was supplied with water ad libitum. Environmental conditions were controlled to provide a temperature of 24 *±* 2°C, a relative humidity of 50 *±* 5%, 100% fresh air at a rate of ≥12 room changes per hour, and a 12:12 h light/dark cycle. Monkeys were given toys. The animals were sacrificed after deep anesthesia using ketamine (20 mg/kg) by intramuscular injection. All mouse investigations were carried out according to the guidelines of the Animal Care and Use Committee of GIST. The protocol was approved by the Animal Care and Use Committee of GIST (permit number: GIST 2011–13). Mice were sacrificed by cervical dislocation.

### Antibodies

For production of polyclonal antibodies, glutathione S-transferase (GST) fusion proteins containing the cytoplasmic tail domains of mouse ADAM2-CyT-1 (amino acids 723–735), mouse ADAM2-CyT-2 (amino acids 721–734), and human ADAM2 (amino acids 709–735), and the cysteine-rich/EGF-like domains of mouse ADAM1B (amino acids 491–697) were expressed in *Escherichia coli* BL21 and affinity purified with glutathione Sepharose 4B (GE Healthcare). The recombinant proteins (GST-ADAM) were used as antigens for producing rabbit polyclonal antisera. An antibody against the human ADAM2 cysteine-rich domain was produced by immunizing rabbits with the relevant peptide (amino acids 576–591) synthesized by custom peptide provider (AbFrontier). The antibodies were affinity purified using the appropriate proteins and an AminoLink immobilization kit (Pierce). An affinity-purified rabbit polyclonal antibody to the cytoplasmic tail domain of mouse ADAM7 was prepared as previously described [25]. The following commercially available antibodies were also used: mouse monoclonal antibodies against ADAM2 (1/1000, MAB19292) and ADAM3 (1/1000, MAB19291) from Millipore; rabbit polyclonal antibodies against ADAM2 (1/100, HPA024621 and HPA026581) from Sigma-Aldrich; an antibody against heat shock protein 5 (HSPA5; 1/1000, H-129) from Santa Cruz; an antibody against protein kinase cAMP-dependent catalytic alpha (PRKACA; 1/1000, ab96186) from Abcam; a monoclonal mouse antibody against protein kinase cAMP-dependent regulatory type I alpha (PRKARIα; 1/1000, 61069) from BD Bioscience; an antibody against α-tubulin (1/1000, T6199) from Sigma-Aldrich; and an antibody against GAPDH (1/1000, MCA4739) from Bio-Rad. As secondary antibodies for the Western blot analysis, we used horseradish peroxidase (HRP)-conjugated anti-rabbit, anti-mouse IgG (Jackson ImmunoResearch) and normal rabbit serum (NRS; Thermo Scientific).

### Preparation of samples

Mouse, monkey and human testes were lysed in nonionic detergent lysis buffer (0.1% Nonidet P-40, 10 mM HEPES, 1.5 mM MgCl_2_, 10 mM KCl, 0.3 mM sucrose, 0.1 mM ethylenediaminetetra-acetic acid, and 25% glycerol) with 1% protease inhibitor cocktail (GenDEPOT) for 1–8 h on ice. Debris was removed by centrifugation at 13,000 g for 15 min at 4°C. The protein concentration of the supernatant was determined by the Bradford method (Bio-Rad). Normal semen (World Health Organization, 2010) was collected from donors who had masturbated into sterile specimen containers after an abstinence period of 48 h, and delivered the samples to the laboratory within 1 h of ejaculation. The semen was allowed to liquefy at room temperature for 30 min, and sperm were separated from the seminal plasma by centrifugation (1500 g) for 15 min at 4°C. The sperm were washed by suspension in phosphate-buffered saline (PBS) and centrifuged at 1500 g at 4°C. The supernatant was discarded and the washing procedure was repeated three times. Monkey sperm were collected from ejaculations induced by lumbar electronic shock under ketamine anesthesia, and washed three times at 1500 g at 4°C for 5 min in PBS. Cells were also prepared from 8-wk-old ICR male mice and *Adam2*^*-/-*^ mice [13]. Mouse cauda epididymidis and vas deferens were directly put into PBS. The cauda epididymis was minced with scissors to allow sperm to swim out of the tissue. The vas deferens was squeezed to push sperm out. After filtration of the sperm suspensions using nylon mesh sheets, sperm cells were washed three times with PBS by centrifugation at 500 g for 5 min at room temperature. The collected sperm were resuspended in 1XSDS sample buffer (3% SDS) and then either boiled for 5 min or lysed with non-reducing detergent (1% NP-40) for 1 h on ice in the presence of protease inhibitors. Each lysate supernatant was mixed with 2XSDS sample buffer (final 3% SDS) and boiled for 4 min. Samples were either left non-reduced or were reduced with 5% β-mercaptoethanol, as indicated in the text and figure legends. To test for dimerization of ADAM2, the protein lysates were treated with a low concentration of SDS at room temperature. In guinea pigs and mice, the ADAM2 complexes were found to be stable in such a condition and detected as distinctive bands in SDS-PAGE followed by western blot analysis [2, 26]. Spermatogenic cells were lysed with 1% NP-40 and the lysate supernatants were mixed with equal volumes of 0.6% SDS sample buffer. The samples were incubated at room temperature (25°C) for 5 min under nonreducing conditions and resolved by 10% SDS-polyacrylamide gel electrophoresis (SDS-PAGE).

### Western blot analysis

A protein lysate containing testicular cells (100–200 μg) or sperm (1.5X10^6^) were loaded in each lane. Proteins were denatured by boiling for 10 minutes in the presence of 3% SDS and 5% β-mercaptoethanol, separated by 8 or 10% SDS-PAGE, and transferred onto polyvinylidene difluoride (PVDF) membranes (0.2 μm; PALL Lifesciences). The membranes were blocked with 5% nonfat dry milk in TBS-T (50 mM Tris-HCl, pH 7.5, 150 mM NaCl [TBS] containing 0.1% Tween-20), and then incubated with primary antibodies at room temperature for 1 h. After three 10-min washes with TBS-T, the membranes were incubated with HRP-conjugated secondary antibodies for 1 h at room temperature. Following three washes with TBS-T, immunoreactive proteins were detected using a chemiluminescence kit (Thermo Scientific).

### Immunoprecipitation

Whole testicular cells from wild-type (WT) and *Adam2*^*-/-*^ mice were lysed in lysis buffer (1% Nonidet P-40, 150 mM NaCl, 50 mM Tris-Cl, 1 mM ethylenediaminetetra-acetic acid) including 1% protease inhibitor cocktail on ice for 1 h. Next, 1 mg of each testis lysate was incubated with 10 μg of anti-mouse ADAM2-CyT-1, anti-mouse ADAM2-CyT-2, or NRS at 4°C under rotary agitation for 4 h. Protein A-sepharose (Amersham Biosciences) was added to the lysate, and the mixture was incubated for 4 h at 4°C with rocking. The beads were washed three times with lysis buffer, and the immune complexes were boiled for 10 min in 1XSDS sample buffer. Total cell lysates and immunoprecipitates were subjected to SDS-PAGE under reducing conditions and then analyzed by Western blotting. Monkey testes were lysed with nonionic detergent lysis buffer (0.1% Nonidet P-40, 10 mM HEPES, 1.5 mM MgCl_2_, 10 mM KCl, 0.3 mM sucrose, 0.1 mM ethylenediaminetetra-acetic acid, 25% glycerol) containing a protease inhibitor cocktail. Total tissue lysates (1 mg) were incubated with 10 μg of anti-hADAM2-CyT antibody and protein A-sepharose for 4 h at 4°C. The protein A beads were washed three times with 0.1% Nonidet P-40 lysis buffer at 4°C and resolved by 8% SDS-PAGE.

### Capillary reverse phase liquid chromatography-tandem mass spectrometry (MS/MS) and data analysis

Proteins immunoprecipitated with anti-human ADAM2-CyT antibody and NRS were digested with trypsin and loaded onto fused silica capillary columns (100 μm i.d., 360 μm o.d.) containing 7.5 cm of 5-μm particle Aqua C18 reverse-phase column material (Phenomenex). The column was placed in-line with an Agilent HP1100 quaternary LC pump and a splitter system was used to achieve a flow rate of 250 nL/min. Buffer A (5% acetonitrile and 0.1% formic acid) and buffer B (80% acetonitrile and 0.1% formic acid) were used to create a gradient over 80 min. The gradient profile was as follows: 3 min of 100% buffer A; a 5-min increase from 0% to 15% buffer B; a 57-min increase from 15% to 55% buffer B; and a 15-min increase from 55% to 100% buffer B. The eluted peptides were directly electrosprayed into an LTQ Ion Trap mass spectrometer (ThermoFinnigan, Palo Alto, CA) via the application of 2.3 kV of DC voltage. One full data-dependent MS scan (400–2,000 m/z) and 10 data-dependent MS/MS scans were used to generate the MS/MS spectra of the eluted peptides. A normalized collision energy of 35% was used throughout the data acquisition period. The MS/MS spectra were compared with those contained within the Mouse IPI protein database (ver.3.31) using the TurboSEQUEST and SEQUEST Cluster Systems (14 nodes). DTAselect was used to filter the search results, and the following Xcorr and delta Cn values were applied to different charge states of peptides with a fully tryptic digested end requirement of 1.8 for singly charged peptides, 2.2 for doubly charged peptides, and 3.2 for triply charged peptides, with a delta Cn value of 0.08 for all charge states. Manual assignments of fragment ions in all filtered MS/MS spectra were used to confirm the protein database search results.

## Results

### Analysis of the ADAM2 cytoplasmic tail domain and ADAM2 complex formation in mice

**Fig 1:**
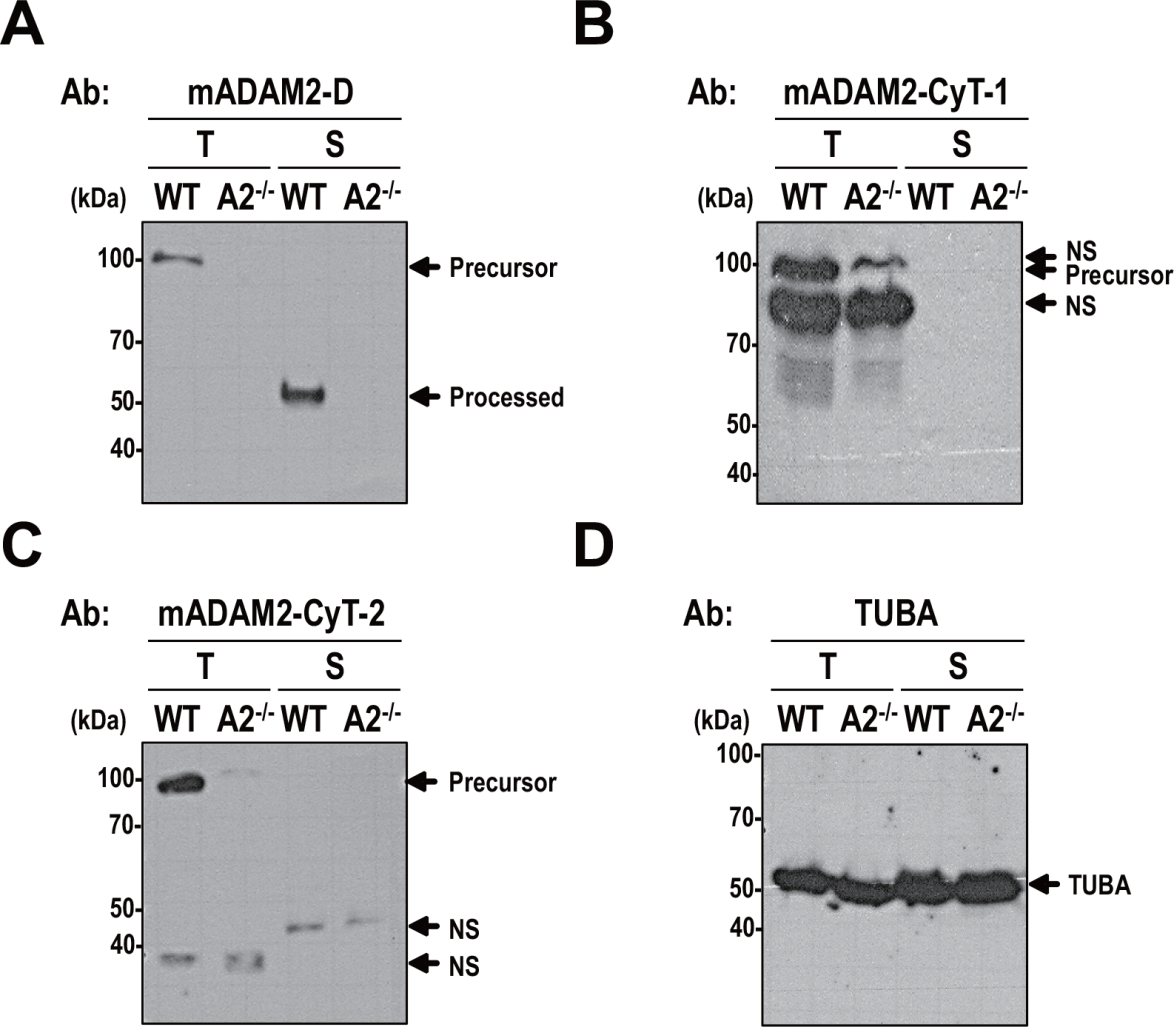
Immunoreactivity of mouse ADAM2 antibodies. Lysates from testicular cells and sperm from wild-type and *Adam2*^*-/-*^ mice were boiled in 3% SDS and 5% β-mercaptoethanol and subjected to Western blot analyses. **A**. Western blotting with the anti-mADAM2-D antibody. This antibody was raised against the ADAM2 disintegrin domain, and recognized the precursor (100-kDa) and processed (45-kDa) forms of ADAM2 in mouse testis and sperm, respectively. **B** and **C**. Blots performed using two different antibodies, which were raised against the cytoplasmic tail domain and designated **B**. anti-mADAM2-CyT-1 and **C**. anti-mADAM2-CyT-2. **D**. An antibody against a-tubulin (TUBA) was included as a control. Experiments were repeated three times. Reduced protein samples were subjected to SDS-PAGE using 10% resolving gels. Abbreviations: T, testicular cells; S, sperm; WT, wild-type; A2^-/-^, *Adam2*^*-/-*^; NS, non-specific. Molecular masses are presented on the left.

It was previously suggested that ADAM2 undergoes modification in its cytoplasmic tail domain during sperm maturation in the epididymis [26]. To further characterize the ADAM2 cytoplasmic domain, we generated antibodies against amino acids 723–735 (mADAM2-CyT-1) and 721–734 (mADAM2-CyT-2) of the mouse ADAM2. Consistent with previous findings obtained using an anti-ADAM2 disintegrin domain antibody (mADAM2-D) that recognized mouse ADAM2 [21] (Fig 1A), both anti-mADAM2-CyT-1 and anti-mADAM2-CyT-2 recognized a 100-kDa band in the testis of WT but not *Adam2*^*-/-*^ mice (Fig 1B and 1C). Anti-mADAM2-CyT-1 did not detect the processed form of ADAM2 in mature sperm (Fig 1B). The 110-kDa and 85-kDa proteins detected by anti-mADAM2CyT-1 in the testis of WT and *Adam2*^*-/-*^ mice are non-specific (Fig 1B). The anti-mADAM2CyT-2 also detected non-specific, 40-kDa bands in both WT and *Adam2*^*-/-*^ testes (Fig 1C). Although anti-mADAM2-CyT-2 detected a faint band between 45 and 50 kDa in WT, this band also was detected in *Adam2*^*-/-*^ sperm (Fig 1C). However, anti-mADAM2-D clearly detected the processed form (45 kDa) of ADAM2 in mature sperm (Fig 1A). The anti-TUBA antibody, which was used as a control, showed that similar amounts of protein were loaded in each lane (Fig 1D). Our results confirm the previous report [26] that an anti-ADAM2 cytoplasmic domain antibody can detect the ADAM2 precursor but not the mature form, suggesting that the cytoplasmic domain of ADAM2 undergoes proteolysis or posttranslational modification during epididymal maturation of mouse sperm.

**Fig 2:**
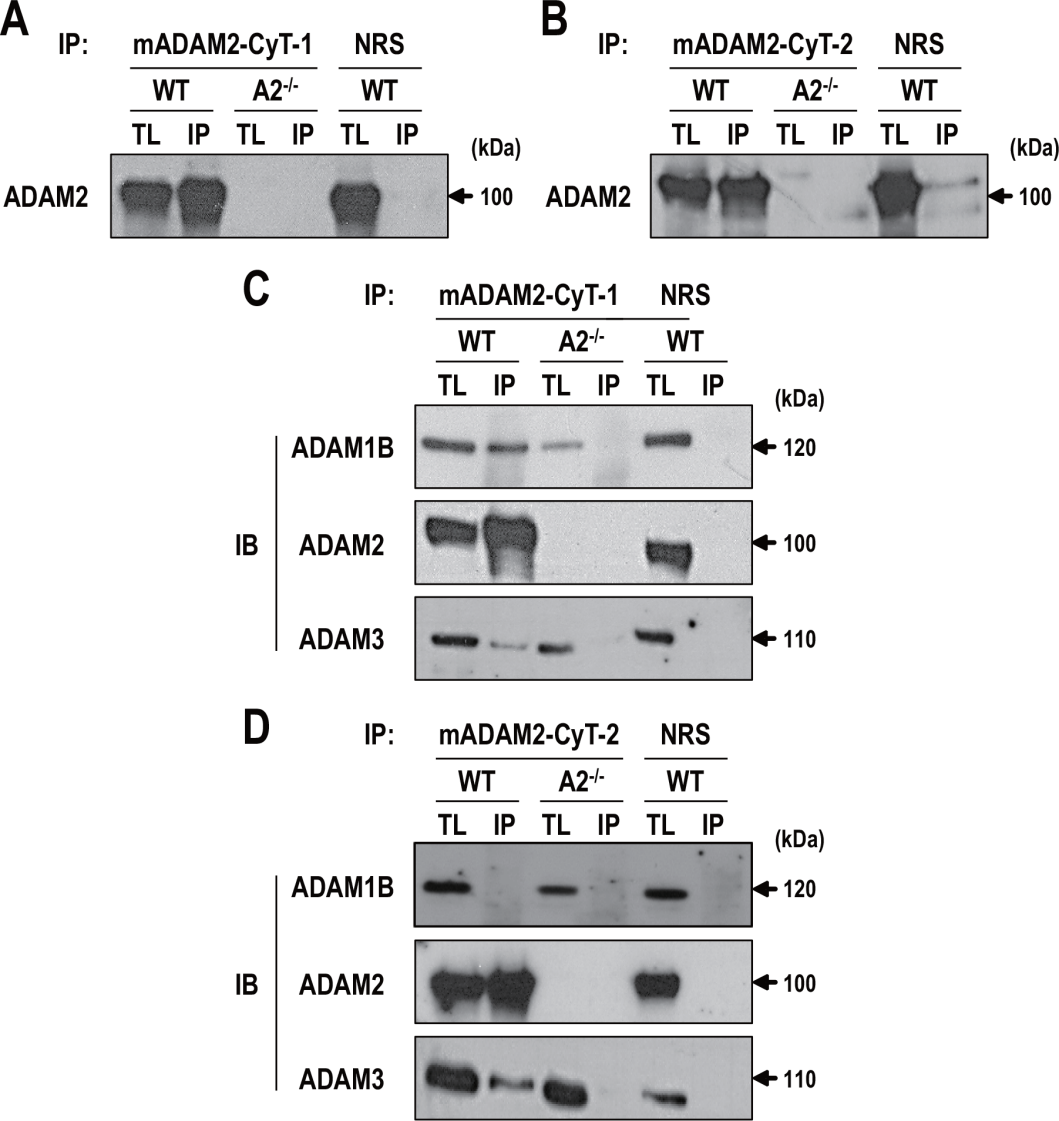
Coimmunoprecipitation of ADAM2 in mouse testes. **A** and **B**. Testes from wild-type and *Adam2*^*-/-*^ mice were lysed with 1% NP-40 buffer, the tissue lysates were immunoprecipitated with **A**. anti-mADAM2-CyT-1 and **B**. anti-mADAM2-CyT-2, and the resolved immunoprecipitates were immunoblotted with anti-mADAM2-D. Normal rabbit serum was used as a control. **C** and **D**. Lysates were precipitated with **C**. anti-mADAM2-Cyt-1 and **D**. anti-mADAM2-CyT-2 and analyzed by immunoblotting with anti-mADAM2-D, anti-mADAM1B, and anti-mADAM3. Experiments were repeated five times. Reduced protein samples were subjected to SDS-PAGE using 8% resolving gels. Abbreviations: WT, wild-type; A2^*-/-*^, *Adam2*^*-/*^; TL, tissue lysate (100 μg); IP, immunoprecipitated protein (1 mg); NRS, normal rabbit serum; and IB, immunoblotting.

ADAM2 is known to assemble into complexes with ADAM1A, ADAM1B, ADAM3, ADAM6 and potentially other ADAMs [15, 17–19]. In an effort to further catalog the ADAM2-associated proteins, we performed coimmunoprecipitation analyses using the anti-mADAM2-CyT-1 and anti-mADAM2-CyT-2 antibodies in mouse testis. Both antibodies were able to effectively precipitate the 100-kDa precursor form of ADAM2 in testis (Fig 2A and 2B). WT testicular protein samples immunoprecipitated with anti-mADAM2-CyT-1 and anti-mADAM2-CyT-2 were immunoblotted with antibodies against ADAM1B, ADAM2-disintegrin, and ADAM3 (no antibody against ADAM1A was available). Testicular protein samples from *Adam2*^*-/-*^ mice were analyzed as a control. Consistent with a previous report [19], WT testicular protein samples immunoprecipitated with the ADAM2 antibodies contained ADAM2 and ADAM3 (Fig 2C and 2D). The ratio of precipitated protein to the total (input) appeared to be lower for ADAM3 than for ADAM2, suggesting that ADAM2-free ADAM3 may be present. We also observed ADAM1B coimmunoprecipitated with ADAM2 when anti-mADAM2-CyT-1 was used (Fig 2C). However, we failed to identify ADAM1B in proteins immunoprecipitated with anti-mADAM2-CyT-2 (Fig 2D). This indicates that the portion of ADAM2 recognized by anti-mADAM2-CyT-2 does not associate with ADAM1B, further suggesting that differential ADAM2 complex formation in the mouse testis is governed (at least in part) by the cytoplasmic tail domain. Alternatively, it is possible that anti-mADAM2-CyT-2 antibody binds the cytoplasmic tail of ADAM2 in the manner which prevents/destabilizes the interaction with ADAM1B.

### Generation of antibodies against human ADAM2

**Fig 3:**
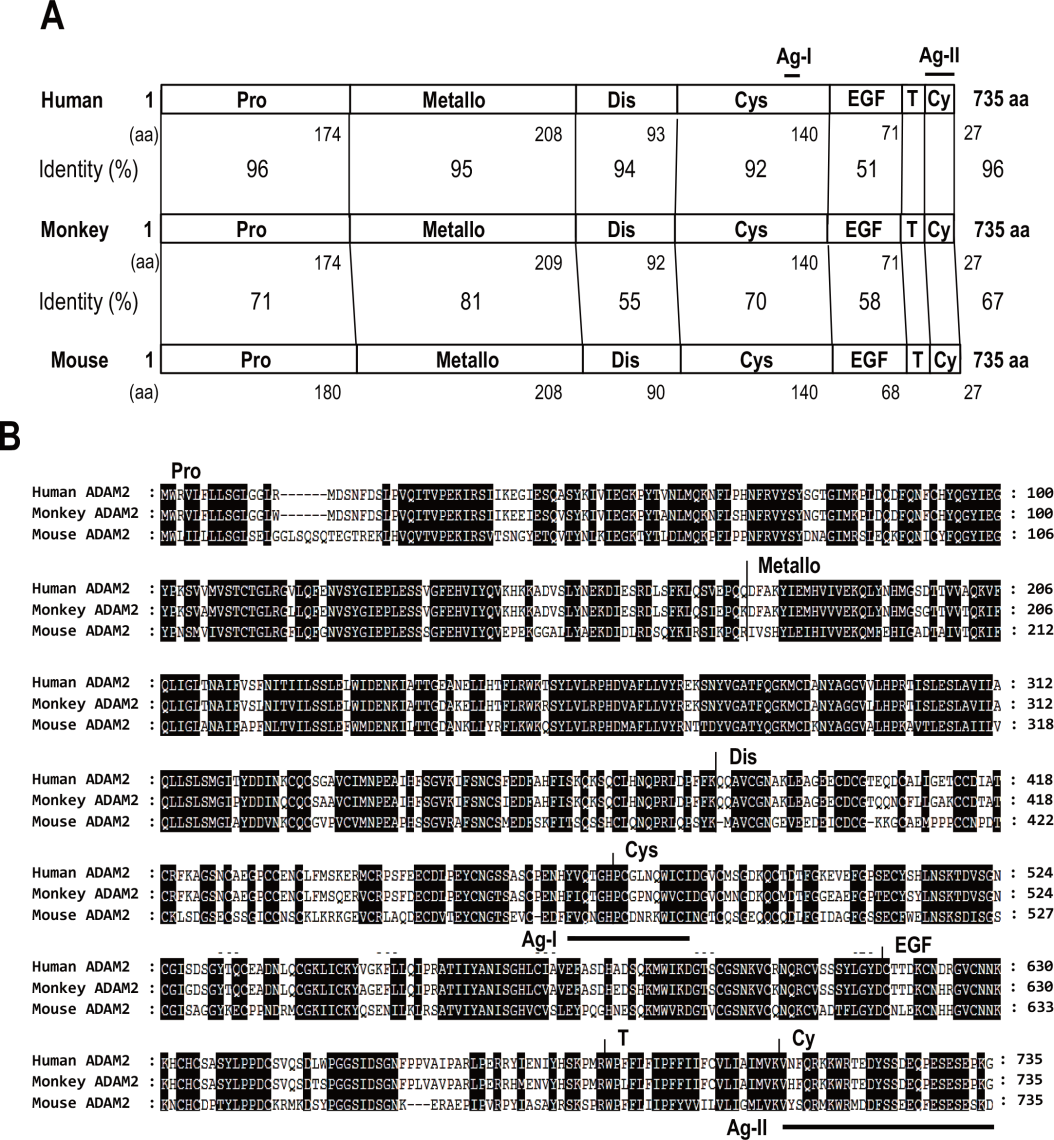
Amino acid sequence comparison of the mouse, monkey and human ADAM2 proteins. **A**. amino acid sequence identities are given as human versus mouse and monkey. **B**. Comparison of amino acid sequences of mammalian ADAM2. The consensus residues are shaded. Abbreviations: Pro, prodomain; Metallo, metalloprotease domain; Dis, disintegrin domain; Cys, cysteine-rich domain; EGF, epidermal growth factor (EFG)-like domain; T, transmembrane domain; Cy, cytoplasmic tail domain; Ag-1, antigen region of the anti-hADAM2-Cys antibody; and Ag-II, antigen region of the anti-hADAM2-CyT antibody.

Although numerous studies have examined ADAM2 in a variety of mammalian species, including mice [2, 3, 13–15, 17–22, 24, 26–35], the protein had not previously been identified or characterized in humans. Comparison of ADAM2 amino acid sequences showed that the mouse, monkey and human sequences exhibited various degrees of identity across different domains (Fig 3). Western blot analysis using the antibodies against mouse ADAM2 did not yield any protein band corresponding to the expected size of the ADAM2 precursor in human testis (data not shown), suggesting that the mouse antibodies were not cross-reactive with human ADAM2. Thus, we raised polyclonal antibodies against the cysteine-rich domain (hADAM2-Cys) and the cytoplasmic tail domain (hADAM2-CyT) of human ADAM2. We also attempted to investigate ADAM2 using commercially available antibodies against human ADAM2, but they were not applicable for Western blot analysis.

**Fig 4:**
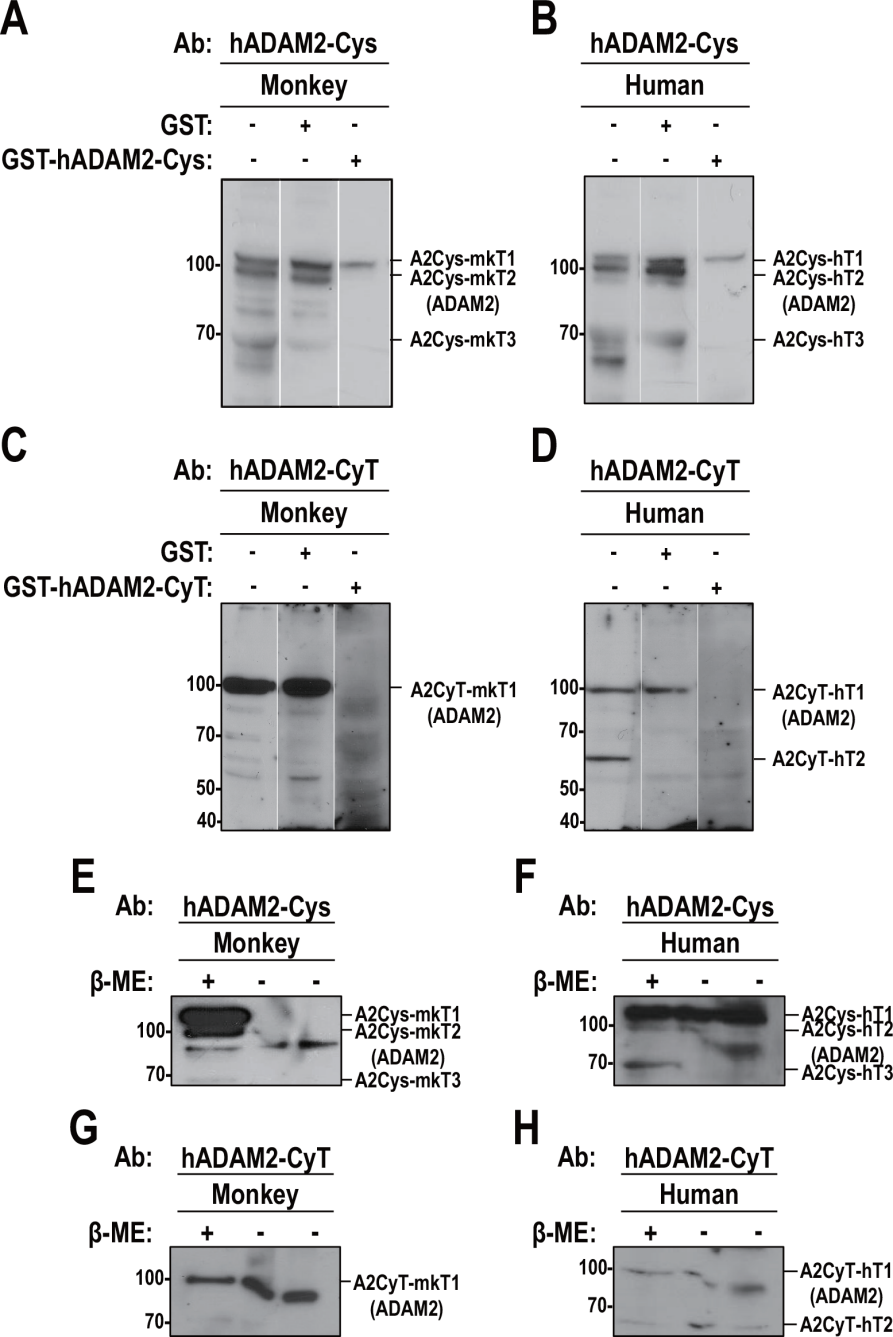
Specificity of the generated antibodies against human ADAM2. **A** and **C**. Testicular germ cells were isolated from monkey testis, boiled in 3% SDS and 5% β-mercaptoethanol, subjected to SDS-PAGE, and blotted with **A**. anti-hADAM2-Cys and **C**. anti-hADAM2-CyT antibodies. **B** and **D**. Human testis was lysed with 0.1% NP-40 buffer, and Western blot analysis was performed using **B**. anti-hADAM2-Cys and **D**. anti-hADAM2-CyT. Glutathione S-transferase or GST recombinant ADAM2 (GST-hADAM2-Cys and GST-hADAM2-CyT) proteins were added to the buffer containing the primary antibodies during Western blot analysis. **E** and **G**. The supernatants from the monkey testis lysates were boiled in 3% SDS with (lane 1) or without (lanes 2 and 3) 5% β-mercaptoethanol, and blotted with **E.** anti-hADAM2-Cys and **G**. anti-hADAM2-CyT. **F** and **H** Human testis supernatants were boiled in 3% SDS and with (lane 1) or without (lanes 2 and 3) 5% β-mercaptoethanol, and immunoblotted with **F**. anti-hADAM2-Cys and **H**. anti-hADAM2-CyT. Experiments were repeated three times. Reduced protein samples were subjected to SDS-PAGE using 8% resolving gels. Abbreviation: GST, glutathione S-transferase; β-ME, β-mercaptoethanol. Molecular masses are presented on the left.

To test the specificity and authenticity of our newly generated antibodies, the corresponding antigens (GST recombinant hADAM2-Cys and hADAM2-CyT proteins) and GST alone were used for Western blot analysis of total proteins from monkey and human testes. These proteins were added during incubation with the anti-hADAM2-Cys or anti-hADAM2-CyT antibodies in the Western blot analysis. Our results revealed that anti-hADAM2-Cys detected bands of 110-kDa, 100-kDa, and 70-kDa in both monkey and human testes; we designate these bands A2Cys-mkT1, A2Cys-mkT2 and A2Cys-mkT3, respectively, in monkey and A2Cys-hT1, A2Cys-hT2 and A2Cys-hT3, respectively, in human. The GST-hADAM2-Cys fusion protein competitively blocked the binding of anti-hADAM2-Cys to A2Cys-mkT2 and A2Cys-mkT3, but not A2Cys-mkT1 in monkey testis (Fig 4A). Similarly, GST-hADAM2-Cys effectively blocked the binding of anti-hADAM2-Cys to A2Cys-hT2 and A2Cys-hT3, but not A2Cys-hT1 in human testis (Fig 4B). Anti-hADAM2-CyT clearly detected the 100-kDa bands in both monkey and human testes (A2CyT-mkT1 and A2CyT-hT1, respectively), and GST-hADAM2-CyT competitively blocked these bindings (Fig 4C and 4D). Although anti-hADAM2-CyT detected the 60-kDa band (A2CyT-hT2) in human testis, this band disappeared when GST or GST-hADAM2-CyT were included during antibody incubation (Fig 4D). These data indicate that the 100-kDa (A2Cys-mkT2, A2Cys-hT2, A2CyT-mkT1 and A2CyT-hT1) and 70-kDa (A2Cys-mkT3, A2Cys-hT3) bands represent potential ADAM2 proteins in monkey and human testes.

The ADAM proteins generally possess numerous cysteine residues [26], meaning that non-reduced proteins migrate faster on SDS-PAGE than reduced proteins. We observed that the molecular sizes of A2Cys-mkT2, A2Cys-hT2, A2CyT-mkT1 and A2CyT-hT1 were decreased under non-reducing conditions (Fig 4E–4H), whereas the other proteins disappeared or remained unchanged. Collectively, these results suggest that the 100-kDa proteins (A2Cys-hT2 and A2CyT-hT1) represented genuine ADAM2 proteins, and that the antibodies generated against human ADAM2 were cross-reactive with monkey ADAM2.

### ADAM2 expression in monkey and human sperm

**Fig 5:**
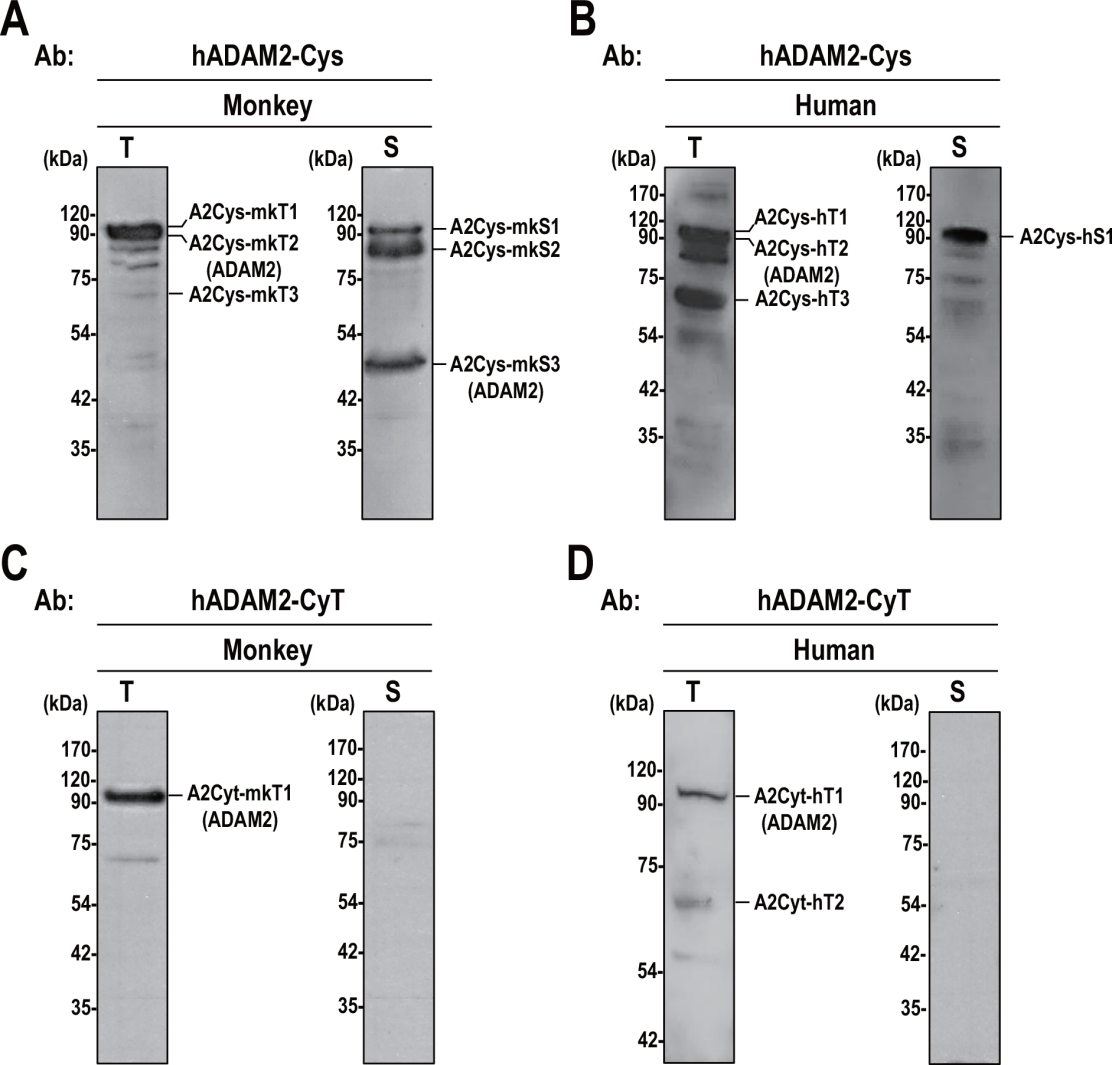
Expression of ADAM2 in monkey and human germ cells. **A** and **B**. Extracts of testicular cells and sperm from **A**. monkeys and **B**. humans were subjected to SDS-PAGE, and blotted with anti-hADAM2-Cys. **C** and **D**. Lysates from **C** monkey and **D** human germ cells were immunoblotted with anti-hADAM2-CyT. Experiments were repeated three times. Reduced protein samples were subjected to SDS-PAGE using 10% resolving gels. Abbreviation: T, testicular cells; S, sperm; and β-ME, β-mercaptoethanol. Molecular masses are presented on the left.

**Fig 6:**
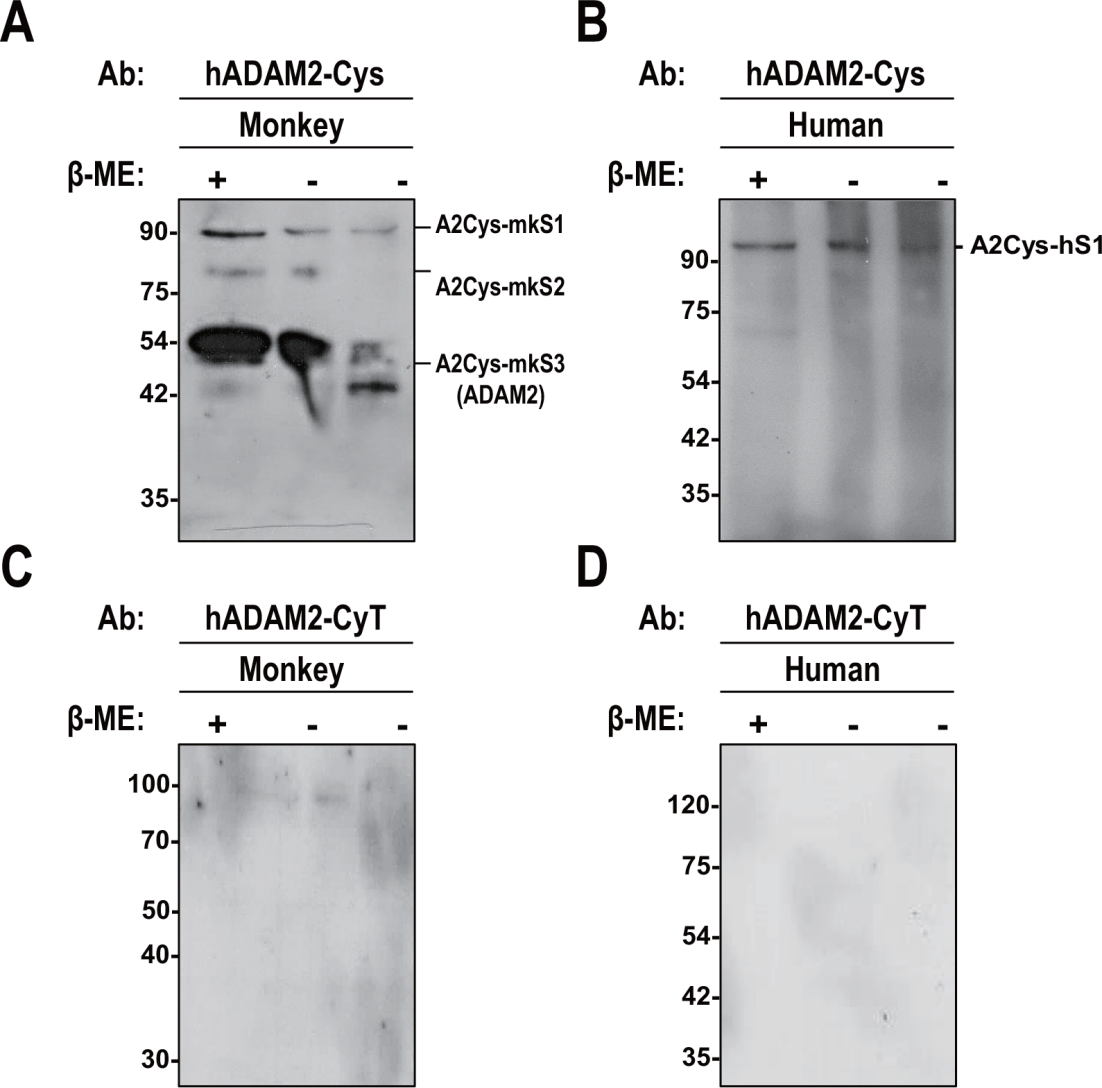
Biochemical characteristics of ADAM2 in monkey and human sperm. **A** and **C.** Monkey sperm were boiled in 3% SDS with (lane 1) or without (lanes 2 and 3) 5% β-mercaptoethanol, resolved by SDS-PAGE and blotted with **A**. anti-hADAM2-Cys and **C**. anti-hADAM2-CyT. The 47-kDa band in the β-mercaptoethanol-free sample was exposed to mercaptoethanol diffusing from the adjacent lane (left). Thus, it is mostly reduced on the left side of the lane and mostly non-reduced on the right side of the lane. **B** and **D**. Human sperm were boiled in 3% SDS with (lane 1) or without (lanes 2 and 3) 5% β-mercaptoethanol, resolved by SDS-PAGE and blotted with **B.** anti-hADAM2-Cys and **D**. anti-hADAM2-CyT. Experiments were repeated twice. Reduced and non-reduced samples were subjected to SDS-PAGE using 10% resolving gels. Abbreviation: β-ME, β-mercaptoethanol. Molecular masses are presented on the left.

To investigate the expression of ADAM2 in monkey and human sperm, we carried out Western blot analyses using the anti-hADAM2-Cys and anti-hADAM2-CyT antibodies, and compared the expression in mature sperm with that in testis. Consistent with the data shown in Fig 4, both antibodies detected ADAM2 (100 kDa) in monkey and human testes (Fig 5A–5D). In monkey sperm, anti-hADAM2-Cys detected a smaller band (47 kDa) that we designated A2Cys-mkS3 (Fig 5A). This is consistent with previous reports on the processed form of ADAM2 in monkey sperm [36, 37]. The apparent molecular size of the 47-kDa protein was decreased under the non-reducing condition (Fig 6A). The antibody also detected the 100-kDa (A2Cys-mkS1) and 88-kDa (A2Cys-mkS2) proteins in monkey sperm (Fig 5A). These proteins were unaltered (A2Cys-mkS1) or undetected (A2Cys-mkS2) under the non-reducing condition (Fig 6A). It is possible that A2Cys-mkS2 could be ADAM2 partially processed, but not recognized by the antibody. The identity of 88-kDa protein needs further investigation. In contrast to anti-hADAM2-Cys, anti-hADAM2-CyT failed to recognize the processed form of ADAM2 in monkey sperm (Figs 5C and 6C). We speculate that monkey ADAM2 may undergo changes in the cytoplasmic domain during sperm maturation, as observed for mouse ADAM2 (Fig 1).

We next used the two anti-human ADAM2 antibodies in Western blot analyses of human sperm. Anti-hADAM2-Cys detected a 110-kDa protein (A2Cys-hS1) in human sperm. The molecular weight of this protein is higher than that of the ADAM2 precursor (A2Cys-hT2), and the band appears to correspond to A2Cys-hT1, which was nonspecifically detected in human testis (Fig 5B). In addition, this band was unchanged under the non-reducing conditions (Fig 6B). Surprisingly, anti-hADAM2-Cys failed to recognize any other distinctive band in human sperm (Figs 5B and 6B). Moreover, anti-hADAM2-CyT did not detect any protein band in human sperm (Figs 5D and 6D). These results suggest that ADAM2 is not expressed in human sperm.

**Fig 7:**
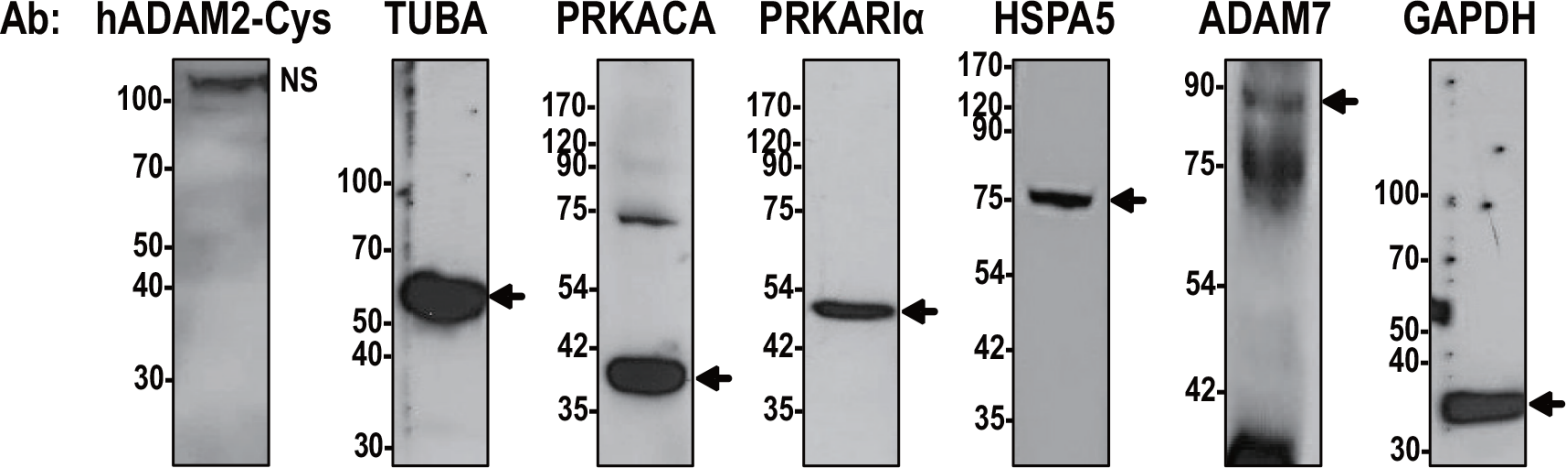
Integrity of human sperm proteins. Western blot analysis was performed using antibodies against ADAM2, PRKACA, PRKARIα, HSPA5, and ADAM7. The TUBA and GAPDH antibodies were used as controls. Experiments were repeated twice. Reduced protein samples were subjected to SDS-PAGE using 10% resolving gels. Abbreviation: NS, non-specific band. Molecular masses are presented on the left.

To ensure the integrity of the human sperm proteins used in the analysis shown in Figs 5 and 6, we carried out Western blot analysis with various antibodies against sperm proteins, including PRKACA, PRKARIα, HSPA5, ADAM7, TUBA, and GAPDH. PRKACA and PRKARIα are the catalytic and regulatory subunits of PKA, respectively, and are known to be present in human sperm [38]. HSPA5 plays an active role in remodeling the sperm surface during capacitation [39]. ADAM7 is synthesized in the epididymis and is known to be secreted and transferred to mature human sperm [40, 41]. All of these marker proteins were detected at their expected molecular sizes (Fig 7), confirming the integrity of the human sperm proteins studied herein.

To further investigate the existence of ADAM2 in human sperm, we compared our findings to the results of previous proteomic studies. Consistent with our Western blot results, ADAM2 was previously identified in monkey testis, monkey sperm, and human testis (Table 1) [42–47]. Although other ADAMs, including ADAM29, ADAM30, and ADAM32, were detected in the previous proteomic analyses of human sperm, ADAM2 was not (Table 1) [48–54]. These data further support our contention that ADAM2 is not expressed in human sperm or at least that it cannot be detected under the conditions used here.

**Table 1:** Previous proteomic analyses.

Human							
Reference	Type of sample	Proteomic approach	Number of proteins described	ADAM2		ADAMs	
				Testis	Sperm	Testis	Sperm
Guo X et al. (2010) [42]	Fertile testis	MALDI-MS/MS	462	No detailed protein list			
Liu M et al. (2013) [44]	Fertile testis	HPLC-MS/MS	7346	+	NA	18, 29, 30, and 32	NA
Li J et al. (2011) [43]	Fertile testis	MALDI-TOF/MS	725	-	NA	29 and 32	NA
Martínez-Heredia J et al. (2006) [51]	Fertile sperm	MALDI-TOF MS MALDI-TOF/TOF-MS	98	NA	-	NA	-
Baker MA et al. (2007) [49]	Fertile sperm	LC-MS/MS	1056	NA	-	NA	29
de Mateo S et al. (2007) [50]	Fertile sperm	MALDI-TOF MS	131	NA	-	NA	-
Siva AB1 et al. (2010) [53]	Fertile sperm	MALDI-MS/MS	75	NA	-	NA	-
Nixon B et al. (2011) [52]	Fertile sperm	LC-MS/MS	124	NA	-	NA	29 and 30
Wang G et al. (2013) [54]	Fertile sperm	LC-MS/MS	4675	NA	-	NA	29 and 30
Baker MA et al. (2013) [48]	Fertile sperm	LC-MS/MS	1429	NA	-	NA	30 and 32
Monkey							
References	Type of samples	Proteomic approach	Number of Proteins described	ADAM2		ADAMs	
				Testis	Sperm	Testis	Sperm
Wang J et al. (2014) [46]	Rhesus macaque testis	HPLC-MS/MS	9078	+	NA	8–10, 18, 29, 30, and 32	NA
Skerget S et al. (2013) [45]	Rhesus macaque epididymal sperm	LC-MS/MS	1247	NA	+	NA	1, 10, 30, and 32
Zhou T et al. (2015) [47]	Rhesus macaque ejaculated sperm	LC-MS/MS	2044	NA	+	NA	29, 30, and 32

NA, not applicable.

### Identification of ADAM2-associated proteins in monkey testis

**Fig 8:**
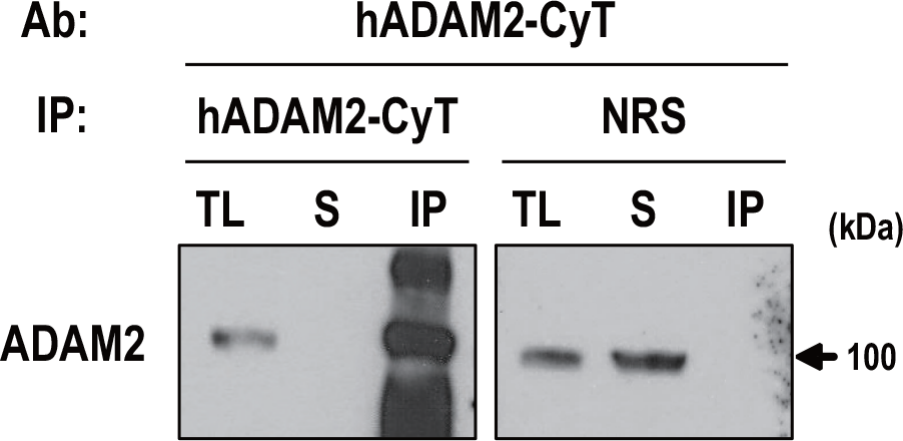
Immunoprecipitation with anti-hADAM2-CyT. Immunoprecipitation of ADAM2 was carried out using monkey testis lysates. Immunoprecipitation using normal rabbit serum was performed as a negative control. Immunoprecipitated lysates were immunoblotted with anti-hADAM2-CyT. Experiments were repeated three times. Reduced protein samples were subjected to SDS-PAGE using 8% resolving gel. Abbreviations: TL, tissue lysate (100 μg); S, supernatant; and IP, immunoprecipitated protein (1 mg); and NRS, normal rabbit serum.

In the course of analyzing the immunoreactivity of the anti-human ADAM2 antibodies, we carried out immunoprecipitation analyses using lysates from monkey testis. We were unable to obtain sufficient lysates from human testis due to limited sample availability. Anti-hADAM2-CyT effectively precipitated the ADAM2 protein from monkey testis lysates (Fig 8), whereas anti-hADAM2-Cys did not (data not shown), indicating that the latter antibody failed to recognize the native form of ADAM2. To investigate whether the ADAM2 is associated with other proteins in monkey testis, the anti-hADAM2-CyT immunoprecipitates were subjected to proteomic analysis by LC-MS/MS (n = 1) (Table 2). NRS was used as a negative control. Commonly detected proteins between anti-hADAM2-CyT and NRS were omitted in Table 2. Our results demonstrated that the precipitates contained ADAM2. Interestingly, this experiment further suggested that uncharacterized protein C19orf71 homolog, 78-kDa glucose-regulated protein (G8F3Q7), and heat shock 70-kDa protein 1-like (HS71L) could be potential ADAM2-interacting proteins (Table 2).

**Table 2:** Monkey ADAM2-coimmunoprecipitated proteins identified by LC-MS/MS analysis.

No	Protein description	Swiss-Prot (UniProtKB)	Matched peptide^a^^)^	Xcorr/DeltaCn (Max)^b^^)^	Measured MW (kDa)/pI^c^^)^	Protein coverage (%)^d^^)^	Spectrum count	Peptides
1	Disintegrin and metalloproteinase domain-containing protein 2	ADAM2_MACFA (spQ28478)	3	4.54/0.58	82.3/6.4	5.4	9	K.SVAMVSTCTGLR.G K.YAGEFLLQIPR.A R.TEDYSTDEQPESESEPK.G
2	Uncharacterized protein C19orf71 homolog	CS071_MACFA (spA5LFW8)	1	2.71/0.74	24.4/9.4	4.3	23	R.WGSTLWKDR.P
3	78-kDa Glucose-regulated protein	G8F3Q7_MACF (trG8F3Q7)	3	4.20/0.58	72.2/5.2	6.7	6	K.NQLTSNPENTVFDAK.R R.TWNDPSVQQDIK.F K.SQIFSTASDNQPTVTIK.V
4	Heat shock 70 kDa protein 1-like	HS71L_MACFA (spQ4R888)	2	3.33/0.32	70.3/6.3	4.2	6	K.VEIIANDQGNR.T R.TPEVTCVVVDVSQEDPDVK.F

a) Number of nonredundant peptides identified for each protein.

b) Maximum Xcorr and DeltaCn values, as derived using the TurboSEQUEST software for any peptide identified for a given protein.

c) Calculated using the TurboSEQUEST software.

d) Protein coverage was calculated by amino acid count.

### Possible ADAM2 complexes in monkey and human testes

**Fig 9:**
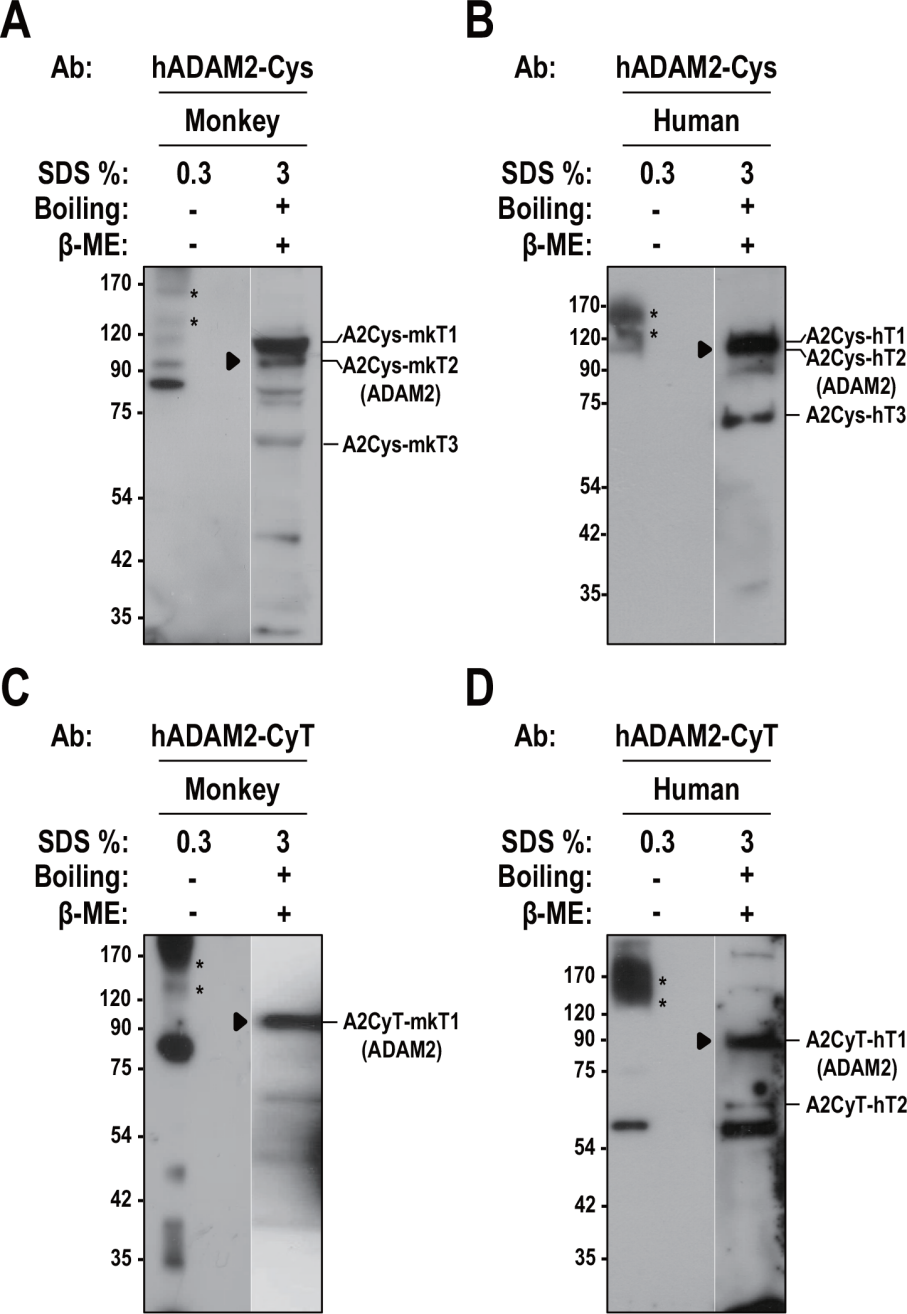
Possible ADAM2 complexes in monkey and human testicular cells. **A** and **C.** The non-reduced supernatants from the lysates of monkey testis were either boiled in 3% SDS (lane 2) or incubated at room temperature in 0.3% SDS (lane 1), and then immunoblotted with **A.** anti-hADAM2-Cys and **C.** anti-hADAM2-CyT. **B** and **D.** A similar experiment was performed using extracts from human testis immunoblotted with **B.** anti-hADAM2-Cys and **D.** anti-hADAM2-CyT. Asterisks and arrowheads indicate the ADAM2 complexes and monomers, respectively. Experiments were repeated three times. Reduced and non-reduced potein samples were subjected to SDS-PAGE using 8% resolving gels. Abbreviation: β-ME, β-mercaptoethanol. Molecular masses are presented on the left.

To examine whether ADAM2 coimmunoprecipitates with other proteins in monkey and human testes, we used the generated antibodies to examine the proteins under mildly denaturing conditions. Consistent with the results shown in Figs 4 and 5, anti-hADAM2-Cys and anti-hADAM2-CyT specifically recognized the 100-kDa ADAM2 protein in monkey and human testes under reducing conditions (Fig 9A–9D). Under non-reducing conditions, ADAM2 was detected as an 80-kDa protein in monkey and human testes (Fig 4E–4H). When gels were run with a low (0.3%) concentration of SDS without pre-boiling of the samples, the 80-kDa ADAM2 protein and additional bands with higher molecular weights (140 and 160 kDa) were seen in both monkey and human testes (Fig 9A–9D). A similar 160-kDa protein was previously detected in monkey testis under similar conditions [37]. These data suggest that ADAM2 may form complexes in monkey and human testes.

## Discussion

In the present study, we investigated and characterized mouse, monkey, and human ADAM2 proteins. In mice, the anti-mADAM2-CyT-1 and anti-mADAM2-CyT-2 antibodies, which were raised against the 13 and 14 carboxy-terminal residues of the cytoplasmic tail domain, respectively, detected the ADAM2 precursor (100-kDa) but not the processed form of the protein (45-kDa). A previous report suggested that mouse ADAM2 is processed or modified at its carboxy-terminal region during epididymal maturation [26]. The modification of the cytoplasmic domain could occur through proteolysis or posttranslational modification. We herein show that the cytoplasmic domain is relevant to the association of ADAM2 with ADAM1B, providing the first evidence that the cytoplasmic domain of ADAM2 may be involved in forming complexes. Despite the small amino acid differences between the immunogens used to raise the anti-mADAM2-CyT-1 and anti-mADAM2-CyT-2 antibodies, ADAM2 binding to the ADAM1B was remarkably different between the antibodies. We do not know how the cytoplasmic domain contributes to mediating complex formation. Further work is needed to elucidate this mechanism and the function of the cytoplasmic domain during complex formation.

We analyzed monkey and human ADAM2 with the antibodies we had newly generated against human ADAM2. In mice, ADAM2 is present as a large precursor (100-kDa) in the testis and is subsequently processed to a mature form (45-kDa) through the removal of N-terminal domains during the maturation of sperm in the epididymis [26]. Similarly, we found that monkey ADAM2 is present as a large precursor (100-kDa) in the testis and as a processed form (47-kDa) in mature sperm. This is also consistent with the previous studies on monkey ADAM2 [37]. As seen for antibodies against the cytoplasmic domain of mouse ADAM2, the anti-hADAM2-CyT antibody did not detect the processed form in monkey sperm, suggesting that the cytoplasmic domain of monkey ADAM2 is modified during sperm maturation. Notably, when we immunoprecipitated protein lysates from monkey testis with the anti-hADAM2-CyT antibody, the precipitates did not contain other ADAM proteins, besides ADAM2. Instead, this analysis identified interactions with the heat shock protein family members, G8F3Q7 and HS71L, suggesting that ADAM2 forms chaperone complexes in monkey testis. Molecular chaperones are critically involved in spermatogenesis and sperm maturation. In particular, male germ cell-specific chaperones are important in sperm development and transformation into functional sperm [55–60].

We found that human ADAM2 exists as a 100-kDa protein in testis. Among the testicular cells, ADAM2 is likely to be expressed by spermatogenic cells, as the Human Protein Atlas (www.Proteinatlas.org) database shows immunohistochemical staining of ADAM2 in spermatids. Despite the presence of ADAM2 in human spermatogenic cells, however, our Western blot analysis did not detect ADAM2 in human sperm. Our findings are consistent with previous proteomic studies that failed to detect ADAM2 in human sperm (Table 1). We do not know the molecular mechanism that might account for the retention of ADAM2 within the testis. Lack of testicular ADAMs in mature sperm is rare, but not impossible. In mice, some ADAM proteins, such as ADAM1A, ADAM21, and ADAM29, are synthesized in the testis, but are absent from mature sperm [17, 24, 61].

As seen for human ADAM2, zonadhesin (ZAN) [45] is found in monkey and mouse sperm but not in human sperm. This could suggest that such a protein experienced a unique evolutionary trajectory in humans. The mammalian genes that encode sperm proteins undergo rapid evolution, with the selective pressures that arise from sperm competition or sperm-egg interactions yielding positive selection for certain protein functions [62]. In rodents, sperm morphology with hook-shaped head could be explained by selective pressures driving sperm toward forming a stronger attachment for fertilization. In primates, the selective pressures generated by sperm competition tend to produce longer, more motile sperm [47]. Interestingly, the disintegrin domain of ADAM2 has been found to be under accelerated evolution in humans [63]. The absence of ADAM2 from human sperm may reflect that, unlike in mice, the protein is no longer required for sperm competition or function in humans. However, the ADAM2 present in testicular germ cells could indirectly contribute to the functions of human sperm. In mice, a complex formed between ADAM2 and ADAM1A in testicular cells is known to regulate the integrity of ADAM3, which is critical for sperm functions [15]. In humans, *ADAM1* is a pseudogene and *ADAM3* is a non-functional gene [1]. Thus, we speculate that human ADAM2 could associate with a yet-unknown protein in spermatogenic cells to regulate fertilization-related sperm proteins. In this regard, it seems noteworthy that our analyses revealed the presence of ADAM2 chaperonic complexes in monkey testis, and a potential ADAM2 complex involving yet-unknown proteins in human testis.

In sum, we herein characterized ADAM2 in mouse, monkey, and human germ cells and report new information for the ADAM2 proteins of each species. We observed species-level differences in ADAM2 expression, suggesting that this protein plays different reproductive functions across mammalian species.
